# Camphor as a Preservative of Urine

**Published:** 1914-06

**Authors:** 


**Camphor as a Preservative of Urine.** Jacob Rosenblum of Pittsburg, N. Y. Med. Jour., April 11, 1914, after reviewing the well known objections to chloroform, formaldehyd. thymol, etc., advises camphor, 1 gram for a 24-hour specimen.

